# The Impact of Ischemia Assessed by Magnetic Resonance on Functional, Arrhythmic, and Imaging Features of Hypertrophic Cardiomyopathy

**DOI:** 10.3389/fcvm.2021.761860

**Published:** 2021-12-17

**Authors:** Sílvia Aguiar Rosa, Boban Thomas, António Fiarresga, Ana Luísa Papoila, Marta Alves, Ricardo Pereira, Gonçalo Branco, Inês Cruz, Pedro Rio, Luis Baquero, Rui Cruz Ferreira, Miguel Mota Carmo, Luís Rocha Lopes

**Affiliations:** ^1^Department of Cardiology, Santa Marta Hospital, Lisbon, Portugal; ^2^NOVA Medical School, Faculty of Medical Science of Lisbon, New University, Lisbon, Portugal; ^3^Heart Centre, Hospital Cruz Vermelha Portuguesa, Lisbon, Portugal; ^4^Epidemiology and Statistics Unit, Research Centre, Centro Hospitalar Universitário de Lisboa Central and Centre of Statistics and its Applications, University of Lisbon, Lisbon, Portugal; ^5^Department of Cardiology, Hospital Garcia de Orta, Almada, Portugal; ^6^Inherited Cardiac Disease Unit, Bart's Heart Centre, St Bartholomew's Hospital, London, United Kingdom; ^7^Centre for Heart Muscle Disease, Institute of Cardiovascular Science, University College London, London, United Kingdom; ^8^Cardiovascular Centre, University of Lisbon, Lisbon, Portugal

**Keywords:** hypertrophic cardiomyopathy, coronary microvascular dysfunction, cardiovascular magnetic resonance, tissue characteristics, functional capacity, arrhythmia

## Abstract

**Aims:** The aim of the study is to investigate the association between the degree of ischemia due to coronary microvascular dysfunction (CMD) and the left ventricular (LV) tissue characteristics, systolic performance, and clinical manifestations in hypertrophic cardiomyopathy (HCM).

**Methods and Results:** This prospective study enrolled 75 patients with HCM without obstructive epicardial coronary artery disease. Each patient underwent cardiovascular magnetic resonance (CMR) including parametric mapping, perfusion imaging during regadenoson-induced hyperemia, late gadolinium enhancement (LGE) and three-dimensional longitudinal, circumferential, and radial strains analysis. Electrocardiogram, 24-h Holter recording, and cardiopulmonary exercise testing (CPET) were performed to assess arrhythmias and functional capacity. In total, 47 (63%) patients were men with the mean age of 54.6 (14.8) years, 51 (68%) patients had non-obstructive HCM, maximum wall thickness (MWT) was 20.2 (4.6) mm, LV ejection fraction (LVEF) was 71.6 (8.3%), and ischemic burden was 22.5 (16.9%) of LV. Greater MWT was associated with the severity of ischemia (β-estimate:1.353, 95% CI:0.182; 2.523, *p* = 0.024). Ischemic burden was strongly associated with higher values of native T1 (β-estimate:9.018, 95% CI:4.721; 13.315, *p* < 0.001). The association between ischemia and LGE was significant in following subgroup analyses: MWT 15–20 mm (β-estimate:1.941, 95% CI:0.738; 3.143, *p* = 0.002), non-obstructive HCM (β-estimate:1.471, 95% CI:0.258; 2.683, *p* = 0.019), women (β-estimate:1.957, 95% CI:0.423; 3.492, *p* = 0.015) and age <40 years (β-estimate:4.874, 95% CI:1.155; 8.594, *p* = 0.016). Ischemia in ≥21% of LV was associated with LGE >15% (AUC 0.766, sensitivity 0.724, specificity 0.659). Ischemia was also associated with atrial fibrillation or flutter (AF/AFL) (OR-estimate:1.481, 95% CI:1.020; 2.152, *p* = 0.039), but no association was seen for non-sustained ventricular tachycardia. Ischemia was associated with shorter time to anaerobic threshold (β-estimate: −0.442, 95% CI: −0.860; −0.023, *p* = 0.039).

**Conclusion:** In HCM, ischemia associates with morphological markers of severity of disease, fibrosis, arrhythmia, and functional capacity.

## Introduction

Hypertrophic cardiomyopathy (HCM) is defined by unexplained left ventricular (LV) hypertrophy in the absence of abnormal loading conditions (1). In HCM, coronary microvascular dysfunction (CMD) and ischemia have been attributed to reduced capillary density, vascular remodeling, fibrosis, myocyte disarray, and extravascular compression (2, 3).

Myocardial fibrosis is a cardinal feature in HCM, with two patterns identified: replacement fibrosis and diffuse interstitial fibrosis (4, 5). Myocardial late gadolinium enhancement (LGE) by cardiac magnetic resonance (CMR) correlates with replacement fibrosis in HCM (4, 5). Native T1 mapping and extracellular volume (ECV) have shown to be more reliable to assess diffuse interstitial fibrosis (6), correlating with histological interstitial fibrosis in endomyocardial biopsy (7). ECV along with LGE can estimate the total fibrotic burden in patients with HCM. Native T1 also measures intracellular components and may reflect cellular abnormalities (8).

Increased myocardial fibrosis is frequently interpreted as a consequence of long-standing disease, including ischemia due to microvascular abnormalities. However, the association between fibrosis and small vessel disease is not completely established (9). Despite the important clinical implications myocardial ischemia may have, it is not a systematic evaluated parameter for clinical decision making in HCM.

We prospectively assessed CMD in patients with HCM by assessing myocardial perfusion defects using stress CMR and hypothesized that CMD impacts on tissues abnormalities, arrhythmias, and functional capacity. We also aimed to identify clinical and imaging characteristics associated with ischemic burden.

## Materials and Methods

### Study Design and Sample

Multicenter prospective study with recruitment performed in Hospital de Santa Marta, Centro Hospital Universitário de Lisboa Central and Hospital Garcia de Orta, Almada, between December 2017 and August 2020. The CMR studies were performed at Heart Center, Hospital da Cruz Vermelha Portuguesa, and the remaining investigations took place in Hospital de Santa Marta.

The study included consecutive adult patients with HCM seen in a cardiomyopathy clinic who fulfilled the inclusion criteria and gave informed consent. The diagnosis of HCM was performed according to published guidelines (1); more specifically, anatomical inclusion criteria were a wall thickness ≥15 mm in one or more LV myocardial segments in probands or >13 mm in relatives or mutation carriers, measured by any imaging technique (echocardiography, CMR, or computed tomography), in the absence of another cardiac or systemic cause of LV hypertrophy. Genetic testing was performed according to the physicians' judgement for each particular case and was not considered for this study.

Patients with LV ejection fraction (LVEF) <50%, prior septal reduction therapy (myectomy or alcohol ablation), and epicardial coronary artery disease were excluded. Obstructive epicardial coronary artery disease was excluded by invasive coronary angiography or coronary computerized tomography in symptomatic patients or asymptomatic patients older than 40 years. In asymptomatic patients younger than 40 years and without cardiovascular risk factors, it was assumed a low likelihood of obstructive coronary artery disease. The investigation followed the principles outlined in the Declaration of Helsinki. The institutional ethics committee of the NOVA Medical School, Lisbon and Centro Hospital Universitário de Lisboa Central approved the study protocol. All patients provided written informed consent.

### Clinical Evaluation

At baseline clinical visit, all patients were evaluated regarding symptoms and physical examination and underwent a 12-lead electrocardiogram and a comprehensive echocardiogram., following current guidelines (10). Obstructive HCM was defined by a systolic gradient of ≥30 mmHg in the LV outflow tract (LVOT) at rest or after provocative maneuvers.

Maximal symptom-limited treadmill cardiopulmonary exercise testing (CPET) was performed using a modified Bruce protocol. Oxygen uptake (VO_2_), CO_2_ production (VCO_2_), and ventilation (VE) were measured. Peak VO_2_ was analyzed as the percentage of predicted peak VO_2_ according to age and gender. Minute VE/CO_2_ production (VE/VCO_2_) slope was calculated, and peak circulatory power was determined as peak VO_2_ x peak systolic blood pressure. Arrhythmic response to exercise was defined as the presence of repetitive premature ventricular beats, non-sustained or sustained ventricular tachycardia. Abnormal blood pressure response to exercise was defined as a failure to increase systolic pressure by at least 20 mmHg from rest to peak exercise or a fall of >20 mmHg from peak pressure.

Twenty-four-hours Holter recording was performed to document the presence of permanent or paroxysmal supraventricular arrhythmias [atrial fibrillation (AF) or atrial flutter (AFL)] and ventricular tachycardia.

### CMR Acquisition Protocol and Analysis

All subjects underwent CMR performed on a 1.5T system (Sola, Siemens, Erlangen, Germany) and abstained from caffeine for at least 24 h. Using compressed sensing-based sequence, cine images in three long-axis planes and sequential short-axis slices spanning the entire left ventricle from the base to the apex were acquired. Basal, mid, and apical precontrast and postcontrast short axis T1 maps were generated using a modified look locker inversion sequence in a 5(3)3 configuration. Basal, mid, and apical precontrast short axis T2 maps were generated, using a single shot TrueFISP acquisition. The same three slices were used for stress perfusion CMR 90 s after hyperemia induced by regadenoson. Images were acquired apex to base during breath-hold at the first pass of contrast (60 measurements). A gradient echo sequence was used. LGE images were acquired using a breath-held segmented inversion-recovery steady-state-free precession sequence.

Microvascular dysfunction was considered present if a visual perfusion defect was observed. Perfusion defects were considered surrogates for ischemia. For perfusion assessment and semi-quantification, the myocardium was divided into 32 subsegments (16 American Heart Association (AHA) segments subdivided into an endocardial and epicardial layer). Ischemic burden for each patient was calculated based on the number of involved subsegments, assigning 3% of myocardium to each subsegment, as assessed in previous landmark studies (11, 12). Each segment was analyzed for the presence or absence of perfusion defect. Perfusion defects sparing the subendocardium and coincident with LGE were not considered, as subendocardial involvement is mandatory for microvascular dysfunction defects. The LGE was analyzed on a per-segment basis using a signal threshold vs. reference myocardium of ≥6 standard deviation, as previously validated with high reproducibility for HCM (13). Total LGE was expressed as a proportion of LV mass.

Native T1 and postcontrast T1 values of myocardium were measured from the three slices generating T1 maps. ECV was calculated according to the previous published formula (14) using patient haematocrit collected at the clinical evaluation, with an interval of <1 month between the evaluation and CMR study. T2 values of myocardium were measured from the three slices generating T2 maps. For each parameter, all 16 AHA segments were included in the analysis.

Three-dimensional longitudinal, circumferential, and radial strains were obtained using an automatic feature-tracking algorithm.

### Statistical Analysis

An exploratory analysis of the variables under study was carried out with categorical variables being described by frequencies (percentages) and quantitative variables by the mean (standard deviation). Locally weighted scatterplot smoothers were used to study the association between MWT and ischemia and between ischemia and LGE.

To identify factors contributing to ischemia (dependent variable) and to study the association between ischemia (independent variable) and tissue characteristics, LV performance, and clinical manifestations, generalized linear regression models for continuous and binary response were applied. Univariable and multivariable models were included patients' characteristics which might potentially influence these outcomes, including age, gender, cardiovascular risk factors, maximum wall thickness **(**MWT), LV mass, the presence of LVOT obstruction, and other CMR findings.

All variables that in the univariable analysis attained a *p* ≤ 0.25 were selected for the multivariable models. Crude and adjusted odds ratios (OR), and crude and adjusted regression coefficients (β) were estimated with corresponding 95% confidence intervals (95% CI). For the linear regression models, the normality assumption of the residuals was verified using Shapiro–Wilk test. To check the assumption of linearity to the logit for the continuous independent variables in the logistic regression analyses, generalized additive regression models for binary response were used.

Considering the outcome LGE as a binary variable (LGE > 15%; LGE ≤ 15%), a cutoff point was determined for ischemia using the criterion that maximizes sensitivity and specificity.

For LGE, a stratified analysis by some groups (MWT, obstructive HCM, gender, and age) was also performed using linear regression models.

The level of significance α = 0.05 was considered, although *p*-values greater than 0.05 and lower than 0.1 (weak evidence of the difference or association) were still considered.

Data were analyzed using the Statistical Package for the Social Science for Windows, version 25.0 (IBM Corp. Released 2017. IBM SPSS Statistics for Windows, version 25.0. Armonk, NY: IBM Corp.) and R (R: A Language and Environment for Statistical Computing, R Core Team, R Foundation for Statistical Computing, Vienna, Austria, year = 2021, http://www.R-project.org.).

## Results

### Study Population

Seventy-five patients were recruited, mean age 54.6 (14.8) years, 47 (63%) of whom were male. The pattern of hypertrophy was asymmetric septal in 48 (64%), apical in 22 (29%), and concentric in 5 (7%). Fifty-one patients (68%) had non-obstructive HCM and MWT was 20.2 (4.6) mm. An apical aneurysm was noted in four patients, perfusion defect in at least one segment was noted in 68 (91%) patients, ischemic burden was, on average, 22.5 (16.9%) of LV in the overall population, and LGE was detected in 70 (93%) patients. Baseline characteristics are shown in Tables 1, 2.

**Table 1:** Baseline characteristics of the study participants.

	***n* = 75**
Male gender, *n* (%)	47 (63)
Age (years), mean (SD)	54.6 (14.8)
BSA (m^2^), mean (SD)	1.93 (0.21)
Hypertension, *n* (%)	38 (51)
Diabetes, *n* (%)	12 (16)
Dyslipidaemia, *n* (%)	30 (40)
Current smoker, *n* (%)	11 (15)
Family history of HCM, *n* (%)	21 (28)
Beta-blocker, *n* (%)	54 (72)
Calcium channel blocker, *n* (%)	18 (24)
Angiotensin converting enzyme inhibitors, *n* (%)	13 (17)
Angiotensin receptor blockers, *n* (%)	18 (24)
Spironolactone, *n* (%)	3 (4)
Non-obstructive HCM, *n* (%)	51 (68)
NYHA I, *n* (%)	41 (55)
NYHA II-III, *n* (%)	34 (45)
Angina, *n* (%)	25 (33)
Syncope, *n* (%)	2 (3)
Palpitations, *n* (%)	24 (32)

*BSA, body surface area; HCM, hypertrophic cardiomyopathy; NYHA, New York Heart Association; SD, standard deviation*.

**Table 2:** Imaging, arrhythmic, and functional findings.

	***n* = 75**
**CMR findings**	
MWT (mm), mean (SD)	20.2 (4.6)
LV mass indexed (g/m2), mean (SD)	97.2 (30.5)
LVEDV(mL/m2), mean (SD)	60.7 (15.3)
LVESV (mL/m2), mean (SD)	18.3 (8.9)
LVEF (%), mean (SD)	71.6 (8.3)
Ischemia (% of LV), mean (SD)	22.5 (16.9)
Native T1 mapping (ms), mean (SD)	1024.1 (35.8)
ECV (%), mean (SD)	26.7 (4.1)
T2 mapping (ms), mean (SD)	50.5 (2.4)
LGE (% of LV mass), mean (SD)	12.7 (8.6)
Global longitudinal strain (%), mean (SD)	−6.15 (4.79)
Global radial strain (%), mean (SD)	24.26 (9.87)
Global circumferential strain (%), mean (SD)	−17.07 (3.78)
**Arrhythmic findings**	
Sinus rhythm, *n* (%)	59 (79)
AF/AFL, *n* (%)	16 (21)
AF	14
AFL	2
Non-sustained ventricular tachycardia, *n* (%)	17 (23)
**CPET parameters**	
Peak VO_2_ (ml/kg/min), mean (SD)	21.07 (6.68)
predicted peak VO_2_ (%), mean (SD)	84.17 (22.49)
VE/VCO_2_ slope, mean (SD)	29.12 (5.23)
Circulatory power (mmHg.ml/kg/min), mean (SD)	3596.5 (1397.4)
Time to anaerobic threshold (min), mean (SD)	5.9 (3.5)
Peak VO_2_ at anaerobic threshold (ml/kg/min)	14.13 (3.58)

*CMR, cardiovascular magnetic resonance; CPET, cardiopulmonary exercise testing; LV, left ventricular; LGE, late gadolinium enhancement; LVEDV, left ventricular end-diastolic volume; LVEF, left ventricular ejection fraction; LVESV, left ventricular end-systolic volume; SD, standard deviation; VCO_2_, CO_2_ production; VE, ventilation; VO_2_, oxygen uptake*.

Stress perfusion images were interpretable in all patients. LGE images were not interpretable in one patient due to artifact. Also, in one patient, it was not possible to interpret CPET parameters due to intolerance to the face mask leading to inaccurate analysis of expired gases; four patients refused to do CPET.

### Association Between Baseline Clinical and Imaging Characteristics and Ischemic Burden

Clinically relevant baseline characteristics, potentially linked with CMD, were tested for the association with ischemia in patients with HCM (Table 3).

**Table 3:** Univariable linear regression for factors related to ischemia.

	**Univariable**		
	**β-estimate**	**95% confidence interval**	***p*** **-value**
Female	2.084	−6.015 to 10.183	0.610
Age (years)	−0.105	−0.371 to 0.162	0.436
MWT (mm)	1.788	1.033 to 2.542	<0.001
LV mass (g/m2)	0.241	0.124 to 0.358	<0.001
Non-obstructive HCM	−1.132	−9.541 to 7.276	0.789
Hypertension	3.081	−4.736 to 10.898	0.435
Diabetes	2.702	−7.984 to 13.389	0.616
Dyslipidemia	−0.300	−8.310 to 7.710	0.941
Current smoker	3.379	−7.686 to 14.444	0.545
HCM risk-SCD score	1.355	−0.350 to 3.060	0.117

*HCM, hypertrophic cardiomyopathy; LV, left ventricle; SCD, sudden cardiac death*.

**Figure 1:**
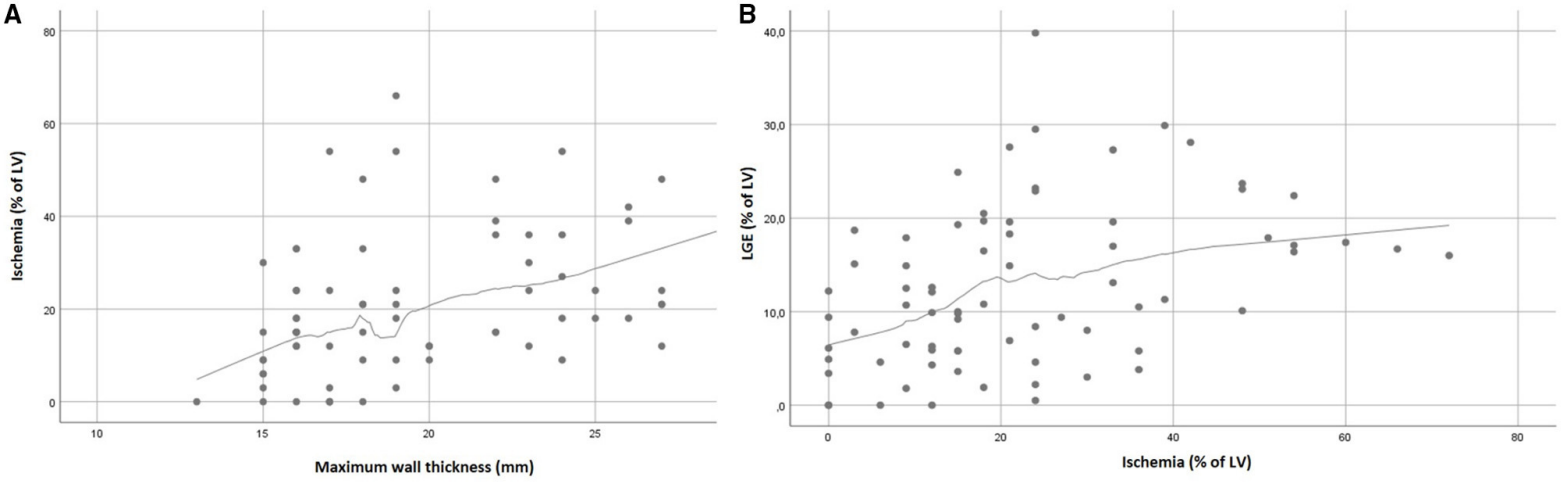
Relationship between MWT and LV ischemia **(A)**, and ischemia and LGE **(B)**.

In univariable analysis, the severity of LV hypertrophy, measured by MWT and LV mass, was associated with more extensive ischemia (MWT β-estimate:1.788, 95% CI:1.033; 2.542, *p* < 0.001; LV mass β-estimate:0.241, 95% CI: 0.124; 0.358, *p* < 0.001). No difference in ischemic burden was found between patients with non-obstructive and obstructive HCM. Demographic factors or cardiovascular risk factors such as diabetes and hypertension were not associated with ischemia. In the multivariable analysis, only MWT is associated with ischemia with a mean increase in LV ischemia of 1.79% for each 1 mm increase in MWT. The association between MWT and ischemia is approximately linear and is graphically represented in Figure 1A.

### Association Between Ischemia and Other CMR Parameters

In this section, the univariable analysis considering the relevant clinical and imaging characteristics is shown in the Supplementary Tables S1, S2.

**Figure 2:**
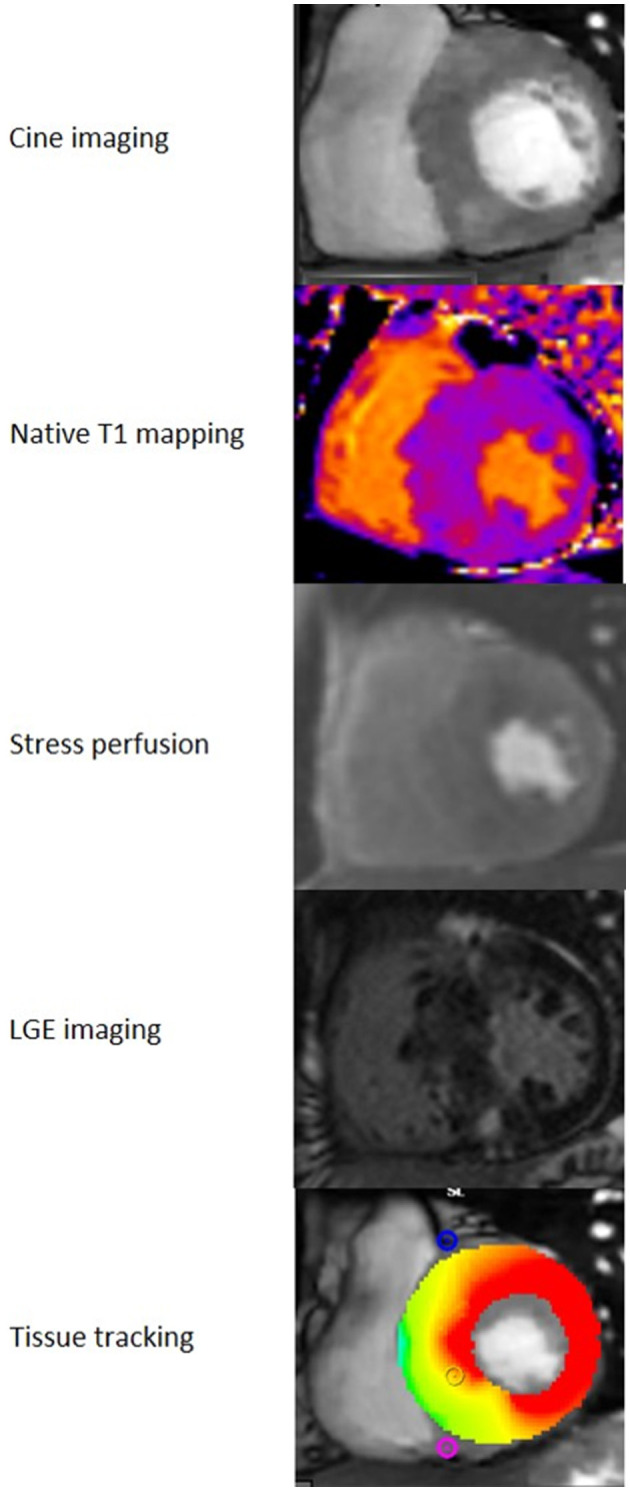
CMR analysis. CMR images from a patient with HCM. Note the asymmetrical septal hypertrophy in the cine images, perfusion defects in the most hypertrophied segments in the stress perfusion acquisition and midwall LGE in the septum and right ventricle insertion points.

CMR images of the different acquisitions are shown in Figure 2.

The extent of ischemia was associated with higher values of native T1 in multivariable analysis (Table 4). For each 10% increase in LV ischemia, there was a mean increase of 9 ms in the value of native T1.

**Table 4:** Multivariable linear regression for factors related to tissue characteristics.

	**Multivariable**		
	**β-estimate**	**95% confidence interval**	***p*-value**
**Native T1 (ms)**			
Ischemia (% of LV)^*^	9.018	4.721; 13.315	<0.001
MWT (mm)	2.569	0.981; 4.157	0.002
**ECV (%)**			
Ischemia (% of LV)^*^	0.002	−0.005; 0.009	0.566
MWT (mm)	0.003	0.001; 0.005	0.016
**LGE (%of LV)**			
Ischemia (% of LV)^*^	1.070	−0.106; 2.245	0.074
MWT (mm)	0.716	0.276; 1.155	0.002
Subgroup analysis			
MWT 15–20 mm			
Ischemia (% of LV)^*^	1.941	0.738; 3.143	0.002
LV mass (g/m^2^)	0.122	0.012; 0.231	0.031
MWT ≥21mm			
Ischemia (% of LV)^*^	0.050	−2.213; 2.314	0.964
Nonobstructive HCM			
Ischemia (% of LV)^*^	1.471	0.258; 2.683	0.019
MWT (mm)	0.553	0.068; 1.038	0.026
Obstructive HCM			
Ischemia (% of LV)^*^	−1.073	−4.161; 2.015	0.476
MWT (mm)	1.284	0.307; 2.262	0.013
Male			
Ischemia (% of LV)^*^	0.436	−1.287; 2.159	0.613
MWT (mm)	0.939	0.312; 1.566	0.004
Female			
Ischemia (% of LV)^*^	1.957	0.423; 3.492	0.015
Age <40years			
Ischemia (% of LV)^*^	4.874	1.155; 8.594	0.016
MWT (mm)	1.380	0.608; 2.153	0.003
Age ≥40 years			
Ischemia (% of LV)^*^	1.145	−0.141; 2.431	0.080
MWT (mm)	0.515	−0.004; 1.034	0.052
T2(ms)			
Ischemia (% of LV)^*^	0.280	−0.010; 0.580	0.061
Non-obstructive HCM	−1.294	−2.389; −0.199	0.021

*ECV, extracellular volume; HCM, hypertrophic cardiomyopathy; LGE, late gadolinium enhancement; LV, left ventricle; MWT, maximum wall thickness*.

* *For each 10% increment of ischemia*.

Ischemia had a weak evidence of association with ECV in univariable analysis (Supplementary Table S1), and no association was found after adjusting for baseline characteristics (*p* = 0.566).

Higher ischemic burden was associated with the extent of LGE in univariable analysis (β-estimate:2.02, 95% CI:0.93; 3.10, *p* < 0.001) (Figure 1B). Discretizing LGE into a binary variable (LGE > 15%; LGE ≤ 15%), higher ischemia values were obtained for LGE >15%, with an area under the ROC curve of 0.766 (Supplementary Figure S1). At the ischemia estimated cutoff point at 21%, a sensitivity of 0.724 and a specificity of 0.659 were achieved. In the multivariable analysis, ischemia showed only a weak evidence of association with LGE (β-estimate:1.070, 95% CI: −0.106; 2.245, *p* = 0.074) (Table 4).

**Figure 3:**
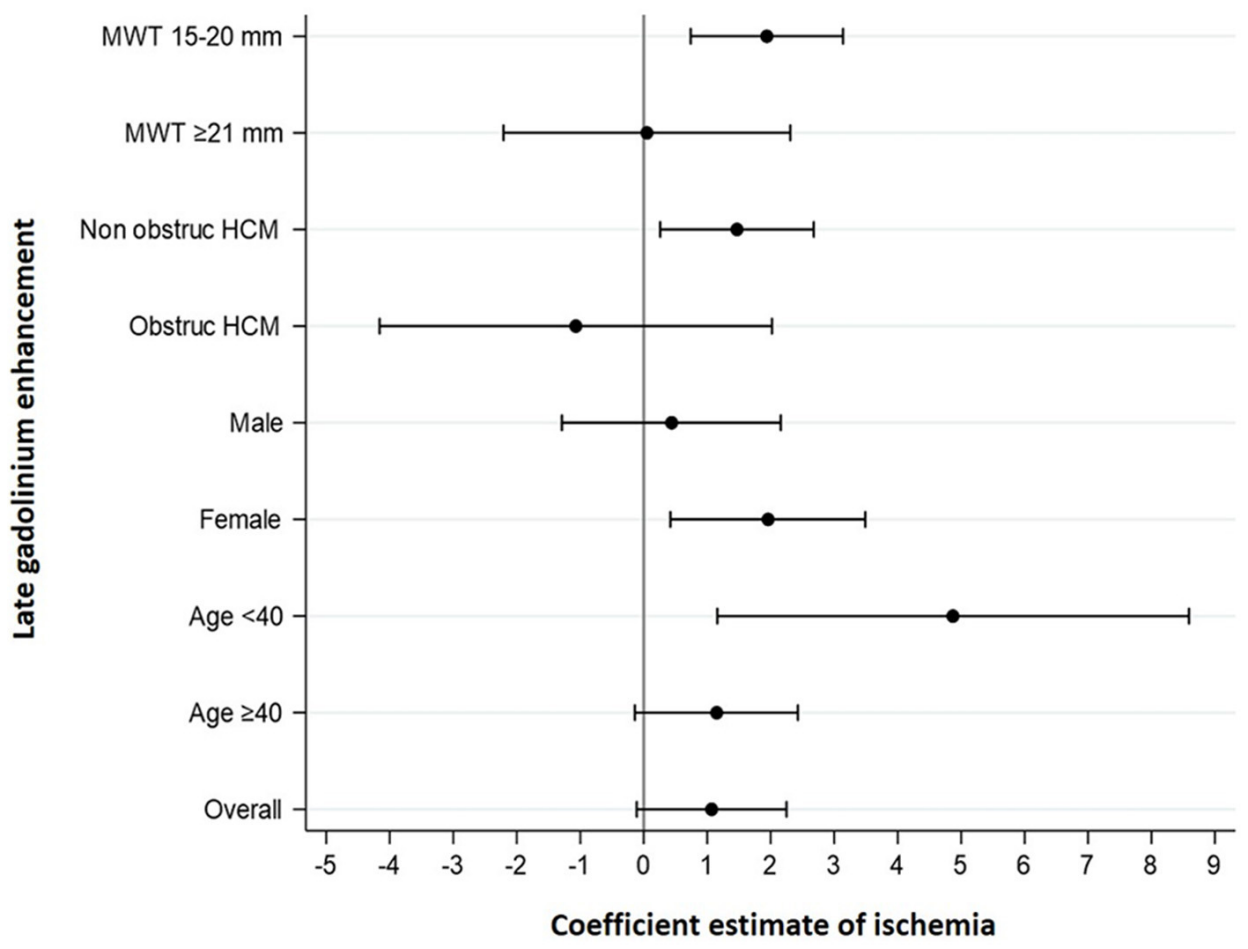
Forest plots for the association between ischemia and LGE. The severity of ischemia was associated with the extent of LGE in patients with MWT 15–20 mm, nonobstructive HCM, women, and age <40 years. Coefficients' estimates and corresponding 95% confidence intervals were obtained by multivariable linear regression models for LGE. MWT, maximum wall thickness; obstruct HCM, obstructive hypertrophic cardiomyopathy.

Because LGE is an important prognostic marker of the disease, a subgroup analysis was further performed to clarify the association between ischemia and LGE. This association was found in individuals with MWT 15–20 mm, non-obstructive HCM, women and age <40 years (Table 4, Figure 3).

In multivariable analysis, ischemia had a weak evidence of association with T2 (β-estimate:0.280, 95% CI: −0.010; 0.580, *p* = 0.061) (Table 4).

Among the other imaging findings, MWT was most prominently related to tissue characteristics, associated with increased values of native T1, ECV, and LGE (Table 4).

In univariable analysis, ischemia was associated with impaired global longitudinal strain (β-estimate: 0.085, 95% CI: 0.020; 0.151, *p* = 0.011) and global radial strain (β-estimate: −0.169, 95% CI: −0.304; −0.034, *p* = 0.015). In multivariable analysis, only LV mass and MWT were independently associated with impairment in myocardial deformation parameters (Supplementary Table S2).

### Impact of Ischemia on Arrhythmias and Functional Capacity

In this section, the univariable analysis considering the relevant clinical and imaging characteristics is shown in the Supplemental Tables S3, S4.

Supraventricular arrhythmias were documented in 16 patients (21%) of whom 6 had permanent AF, 8 paroxysmal AF, and 2 paroxysmal AFL on ECG or Holter monitoring. NSVT was reported in 17 patients (23%), on Holter monitoring.

During CPET, two patients had NSVT and 12 had premature ventricular beats during exercise or recovery phase; four patients presented abnormal BP response.

Regarding ECG abnormalities, namely voltage criteria for LV hypertrophy, QRS fragmentation, and the presence of Q wave and T wave inversion, ischemia was only independently associated with voltage criteria for LV hypertrophy (OR: 1.072, 95% CI: 1.013; 1.135, *p* = 0.017) (Supplementary Table S3).

Ischemia (OR:1.669, 95% CI: 1.082; 2.574, *p* = 0.021) and left atrial volume (OR:1.105,95% CI:1.050; 1.164, *p* < 0.001) were independently associated with supraventricular arrhythmias, mainly AF. No association was verified between ischemia and NSVT (Table 5).

**Table 5:** Multivariable logistic and linear regression for factors related to arrhythmias and functional capacity.

	**Multivariable**		
	**OR estimate**	**95% confidence interval**	***p*-value**
**AF/AFL**			
Ischemia (% of LV)^*^	1.669	1.082; 2.574	0.021
Left atrial volume (ml/m^2^)	1.105	1.050; 1.164	<0.001
**Nonsustained ventricular tachycardia**			
Ischemia (% of LV)^*^	0.864	0.571; 1.307	0.489
Non-obstructive HCM	0.196	0.052; 0.733	0.015
LGE (% of LV)	1.096	1.004; 1.197	0.041
	β-estimate	95% confidence interval	*p*-value
**Peak VO**_**2**_			
Ischemia (% of LV)^*^	−0.170	−0.887; 0.547	0.637
Female	−4.907	−7.580; −2.234	<0.001
Age (years)	−0.202	−0.290; −0.114	<0.001
**Predicted Peak VO**_**2**_			
Ischemia (% of LV)^*^	1.084	−2.133; 4.301	0.503
LVEF (%)	0.618	0.007; 1.228	0.047
LGE (% of LV)	−0.815	−1.478; −0.153	0.017
**VE/VCO**_**2**_ **slope**			
Ischemia (% of LV)^*^	−0.102	−0.716; 0.512	0.741
LGE (% of LV)	0.195	0.069; 0.320	0.003
Age (years)	0.147	0.076; 0.217	<0.001
Diabetes	4.423	1.551; 7.295	0.003
**Time to anaerobic threshold**			
Ischemia (% of LV)^*^	−0.442	−0.860; −0.023	0.039
Non-obstructive HCM	−1.727	−3.255; −0.198	0.027
Age (years)	−0.083	−0.135; −0.031	0.002
Diabetes	−2.330	−4.436; −0.225	0.031
**VO**_**2**_ **at anaerobic threshold**			
Ischemia (% of LV)^*^	−0.159	−0.590; 0.271	0.462
Female	−1.916	−3.534; −0.298	0.021
Age (years)	−0.100	−0.153; −0.046	<0.001
**Circulatory power**			
Ischemia (% of LV)^*^	−10.730	−196.200; 174.741	0.908
MWT (mm)	103.970	29.553; 178.387	0.007
LGE(% of LV)	−67.439	−104.494; −30.383	0.001
Female	−887.509	−1502.111; −272.907	0.005
Age (years)	−20.882	−40.255; −1.510	0.035
Beta-blocker	−758.485	−1364.217; −152.753	0.015

*HCM, hypertrophic cardiomyopathy; LGE, late gadolinium enhancement; LV, left ventricle; MWT, maximum wall thickness; OR, odds ratio; VCO_2_, CO_2_ production; VE, ventilation; VO_2_, oxygen uptake*.

* *For each 10% increment of ischemia*.

Regarding CPET parameters, ischemia was associated with shorter time to anaerobic threshold (β-estimate: −0.442, 95% CI: −0.860; −0.023, *p* = 0.039).

Older age (β-estimate: −0.202, 95% CI: −0.290; −0.114, *p* < 0.001) and female gender (β-estimate: −4.907, 95% CI: −7.580; −2.234, *p* < 0.001) were the characteristics associated with lower peak VO_2_ and lower VO_2_ at anaerobic threshold, whereas LVEF (β-estimate: 0.618, 95% CI: 0.007; 1.228, *p* < 0.047) and LGE (β-estimate: −0.815, 95% CI: −1.478; −0.153, *p* = 0.017) were independently associated with predicted peak VO_2_.

Older age and more extensive LGE were associated with higher VE/VCO_2_ slope whereas older age, female gender, and more extensive LGE showed association with worse circulatory power (Table 5).

## Discussion

In this study, we showed that ischemia had a transversal impact through the pathophysiological aspects of HCM, from the tissue characteristics to the clinical manifestations. Increased severity of ischemia was associated with higher values of native T1 and more extensive LGE and increased the risk of AF/AFL. Patients with ischemia reached anaerobic threshold earlier on CPET.

Studies previously published using CMR and positron emission tomography to study CMD in HCM pointed out that CMD is associated mostly with LV hypertrophy and LGE and linked with worse outcome (Supplementary Table S5).

Our study performed a comprehensive assessment of the impact of CMD among important points of the disease pathophysiology.

In HCM, CMD represents an intrinsic feature, being present in various stages of the disease (15). CMD is mainly caused by intrinsic structural abnormalities in intramural small coronary arteries (3) and by extrinsic compression secondary to LV hypertrophy, LVOT obstruction, and diastolic dysfunction. Despite the identification of these pathophysiological factors, it is not clear which patients with HCM will develop functionally significant CMD.

We found greater ischemic burden in patients with more severe LV hypertrophy, without significant association with the presence of LVOT obstruction. The presence of concomitant comorbidities, previously described as associated with CMD, such as hypertension and diabetes (16, 17) was not associated with the presence of ischemia in our cohort.

### Ischemia and Tissue Characteristics

Ischemia and the severity of LV hypertrophy were the major factors associated with tissue changes. Ischemia was associated with slightly increased native T1, but not with ECV. We hypothesize that this observation is due to CMD having a greater association with intracellular changes rather than interstitial diffuse fibrosis. Regarding intracellular compartment, the elevation of native T1 in HCM probably depicts intracellular changes, namely altered calcium cycling and sarcomeric calcium sensitivity, disturbed biomechanical stress sensing, and impaired cardiac energy homeostasis (18). Abnormal development of coronary circulation may be secondary to the exposure of coronary precursors to abnormal mechanical stimuli by mutated cardiomyocytes (19), contributing for the association between intracellular abnormalities and CMD.

Despite a significant association between ischemia and LGE in univariable analysis, with an increase of 2% in the extent of LGE verified for each 10% increase of ischemia, this association became weak when adjusting for MWT, which was the most important independent factor associated with LGE in the overall population. CMR studies have demonstrated that the severity of LV hypertrophy is linked to the extent of LGE (20, 21), and higher MWT is independently associated with increased LGE progression rate during the follow-up (22).

However, in the subgroup analyses, ischemia was strongly associated with the extent of LGE among the individuals with MWT 15–20 mm, non-obstructive HCM, women and age <40 years. The recognition of the association between ischemia and LGE in particular subgroups raises important points. The ischemic burden was independently associated with the extent of LGE in subgroups classically associated with lower risk of sudden cardiac death, such as MWT 15–20 mm and non-obstructive HCM (1). Despite the development of different approaches for risk stratification, sudden cardiac death prediction is still characterized by a significant amount of unpredictability (23). LGE is associated with sudden cardiac death (20, 24); therefore, the recognition of features associated with fibrosis has the potential to improve the accuracy of risk stratification in these subsets of patients. In younger patients, ischemia had strong correlation with LGE, independently of MWT, which could explain, at least in part, the heterogeneity in the extent of fibrosis found among younger patients. The recognition of an important precursor of the interstitial fibrotic process may allow the development of targeted therapies with significant impact on the disease natural history and prognosis. In HCM, women present less MWT than men (25), and this fact may explain the higher preponderance of ischemia in the fibrotic process, as hypertrophy is not so pronounced. Conversely, MWT was the most important factor associated with LGE in men.

Coronary microvascular dysfunction leads to repetitive episodes of ischemia resulting in myocyte death and fibrotic replacement (26). Postmortem studies in patients with HCM showed different phases of ischemic lesions, including an acute phase with coagulative necrosis and neutrophilic infiltrate to postnecrotic replacement-type fibrosis (2). Interstitial fibrosis derives from fibroblast activity that leads to increased numbers and thickness of collagen fiber, arranged in disorganized patterns (27). The two types of fibrosis reflect different phases of the disease. Whereas interstitial fibrosis is associated with myocyte disarray and predominates at the earliest stages, replacement fibrosis is typically acquired later in the natural history of the disease (28). Histologically, the abnormalities in intramural small vessels closely colocalize to the fibrotic scars, but this association is less stronger with interstitial fibrosis (2, 5, 15). Our findings are in accordance with these published features, as we noted a relationship between ischemia and LGE but not with ECV. High-resolution quantitative perfusion analysis demonstrated a higher ischemic burden in patients with LGE (29), and perfusion mapping confirmed that stress myocardial blood flow was lowest in the fibrotic segments (30). Hyperemic myocardial flow is impaired in areas with LGE and its immediate vicinity compared with remote areas (31). These findings suggested that LGE corresponds, at least in part, to replacement fibrosis secondary to CMD and myocardial ischemia.

Taking into consideration that LGE >15% is a marker of worse outcome (24), we found that ischemia ≥21% of LV has good sensitivity and specificity for the association with LGE >15%.

Myocardial edema evaluated by T2-weighted imaging has been described in patients with HCM, associated with signs of advanced disease, such as higher LV mass, lower ejection fraction, and greater LGE extent (32). Furthermore, high T2 was associated with postexercise troponin rise in patients with HCM, identifying a subset of individuals more vulnerable to myocardial injury after a situation of high oxygen demand (33). In our cohort, ischemia had a weak evidence of association with higher T2 values, whereas non-obstructive HCM was associated with lower T2 compared to obstructive HCM.

### CMD, Arrhythmias, and Functional Capacity

Coronary microvascular dysfunction had strong evidence of association with the presence of supraventricular arrhythmia, mainly AF. For each 10% increase in ischemia, there was an increase of 67% in the odds of AF/AFL, and this association was independent of other well-known predictors of AF such as age and left atrial volume or other markers of the disease severity, such as MWT and LVOT obstruction. Although the atrial wall cannot be accurately assessed by CMR, we hypothesize that this link between AF/AFL and ischemic burden may reflect advanced disease, involving atrial myocardium beyond LV myocardium, contributing for atrial dysfunction and arrhythmogenicity. Furthermore, recurrent episodes of ischemia may contribute to LV diastolic dysfunction and consequent increase in atrial pressures and stretch. In HCM, the relationship between CMD and AF has been previously described even in low-risk patients, independently of atrial dimension (34).

Despite the association between CMD and LGE, and the latest with NSVT, ischemia was not directly linked to NSVT in our cohort. Therefore, this proarrhythmic effect seems to be driven by fibrosis *per se*, without significant contribution from its ischemic precursor.

Age, female gender, and LGE were the most important factors associated with worse functional capacity objectively assessed by CPET. Ischemia was only associated with shorter time to anaerobic threshold. Older age and female gender were associated with lower peak VO_2_ and lower VO_2_ at anaerobic threshold, in line with previous studies (35). Lower peak VO_2_ is associated with increased risk of severe symptoms (36). In HCM, women have shown 50% greater risk of progression to advanced heart failure compared with men, which may be explained by greater prevalence of LV outflow obstruction, smaller LV cavity dimensions, and enhanced susceptibility to LV remodeling (25). In our study, when adjusted to age and gender, LGE became an independent factor associated with predicted peak VO_2_, being also associated with higher VE/VCO_2_ slope and lower peak circulatory power. Higher VE/VCO_2_ slope and worse peak circulatory power, and also the extension of fibrosis (5), have been described markers of worse outcome, including progression to heart failure (37). Our nuanced findings reinforce the link between extensive fibrosis and declined functional capacity.

Besides peak VO_2_ and VE/VCO_2_ slope, anaerobic threshold was found to be a predictor of all-cause mortality or progression to heart transplant in patients with HCM (35, 38). Interestingly, we found a relationship between greater ischemic burden and shorter time to anaerobic threshold. Older patients with non-obstructive HCM and diabetic had a shorter time to anaerobic threshold and therefore more severe ischemia.

We found a significant relationship between LGE and functional capacity, whereas the association between CPET parameters and ischemia was less pronounced. Whereas the progression of myocardial fibrosis is documented in patients who development heart failure and systolic dysfunction, CMD may remain unchanged compared with earlier phases of the disease (15).

The association between diabetes and higher VE/VCO_2_ slope and shorter time to anaerobic threshold was likely in relation to the fact that diabetic patients were older and mostly women.

In our sample, older patients had lower MWT, which may explain the association found between higher MWT and greater circulatory power.

### Limitations

An important limitation of our study is the relatively small sample size and low number of events, which limits the achievement of statistical significance in some associations. There was not a validation of tissue characteristics with histological samples. Perfusion defects on CMR are considered as surrogates for ischemia, similar to several other studies in multiple conditions. We decided to adopt a semi-quantitative analysis of ischemia, based on the presence of visual perfusion defects in 32 segments because it was used in a previous landmark study which compared stress CMR with the gold standard of invasive evaluation by fractional flow reserve (11), and it provides an easily applicable and available method for clinical practice. This approach has the inherent limitation of the visual assessment and the total of LV evaluated is 96% (3% for each segment).

Six patients were in AF during CMR scan, which may interfere in the values obtained for parametric mapping.

## Conclusion

In HCM, ischemia is related to the severity of LV hypertrophy and impacts various pathological and clinical features, encompassing tissue abnormalities and arrhythmic events. Our findings highlight the potential additional role of the evaluation of ischemia in the approach of the patients with HCM and their risk stratification.

## Data Availability Statement

The raw data supporting the conclusions of this article will be made available by the authors, without undue reservation.

## Ethics Statement

The studies involving human participants were reviewed and approved by NOVA Medical School, Lisbon Centro Hospital Universitário de Lisboa Central. The patients/participants provided their written informed consent to participate in this study.

## Author Contributions

SA, AF, MM, and LL contributed to conception and design of the study and interpretation of data. SA, BT, AF, RP, GB, IC, PR, LB, and RF contributed to acquisition and analysis or interpretation of data. AP and MA performed the statistical analysis. SA wrote the first draft of the manuscript. All authors contributed to manuscript revision, read, and approved the submitted version.

## Funding

LL is supported by an MRC UK clinical academic partnership award (CARP) MR/T005181/1.

## Conflict of Interest

The authors declare that the research was conducted in the absence of any commercial or financial relationships that could be construed as a potential conflict of interest.

## Publisher's Note

All claims expressed in this article are solely those of the authors and do not necessarily represent those of their affiliated organizations, or those of the publisher, the editors and the reviewers. Any product that may be evaluated in this article, or claim that may be made by its manufacturer, is not guaranteed or endorsed by the publisher.
