# Supplementary material for: The Impact of Ischemia Assessed by Magnetic Resonance on Functional, Arrhythmic, and Imaging Features of Hypertrophic Cardiomyopathy
Source: Front Cardiovasc Med. 2021 Dec 17;8:761860. doi: 10.3389/fcvm.2021.761860 (PMC8718511; doi:10.3389/fcvm.2021.761860)
Supplement: Supplementary file 1 [file Data_Sheet_1.docx]

Supplementary Material

**METHODS**

**Cardiopulmonary exercise testing and Holter monitoring**

Maximal symptom-limited treadmill cardiopulmonary exercise testing (CPET) was performed using a modified Bruce protocol. CPET and recovery period were performed under monitoring with continuous 12-lead electrocardiogram, blood pressure cuff, saturation probe and a face mask to measure respiratory gases. Blood pressure was measured at rest, in each stage, at peak exercise and at the first, third and sixth minute of the recovery phase. Respiratory gases were analysed using the equipment Ergostik, Geratherm®, Cardio Solutions. Oxygen uptake (VO_2_), CO_2_ production (VCO_2_) and ventilation (VE) were measured on a breath-by-breath basis. Patients were encouraged to perform exercise until the VCO_2_/VO_2_ ratio was ≥1.15. Peak VO_2_ adjusted for body mass or for fat-free mass in obese patients (body mass index >30 kg/m^2^) was analysed as the percentage of predicted peak VO_2_ according to age and gender. Minute ventilation/CO_2_ production (VE/VCO_2_) slope, was calculated by automatic linear regression with values obtained during the exercise test. Peak circulatory power was determined as peak VO_2_ x peak systolic blood pressure. Arrhythmic and blood pressure (BP) responses to exercise were evaluated. Arrhythmic response to exercise was defined as the presence of repetitive premature ventricular beats (seven or more ventricular premature beats per minute, ventricular bigeminy or trigeminy), non-sustained or sustained ventricular tachycardia. Non-sustained ventricular tachycardia (NSVT) was defined as ≥3 consecutive ventricular beats at ≥120bpm lasting <30 seconds. Abnormal BP response to exercise was defined as a failure to increase systolic pressure by at least 20 mmHg from rest to peak exercise or a fall of >20 mmHg from peak pressure.

Twenty-four hour Holter recording was performed to document the presence of permanent or paroxysmal supraventricular arrhythmias (>10 seconds of atrial tachycardia, atrial fibrillation (AF) or atrial flutter (AFL)) and ventricular tachycardia (as defined above).

**CMR acquisition protocol and analysis**

All subjects underwent CMR performed on a 1.5T system (Sola, Siemens, Erlangen, Germany) and abstained from caffeine for at least 24 hours. Cardiac localizers were obtained to plan the cardiac axis views. Using Compressed Sensing-based sequence, cine images in 3 long-axis planes and sequential short-axis slices spanning the entire left ventricle from the base to the apex were acquired. Basal, mid and apical pre-contrast and post-contrastshort axis T1 maps were generated using a Modified Look Locker Inversion (MOLLI) sequence in a 5(3)3 configuration, with a voxel size of 2.00x 2.00 x 8 mm. Basal, mid and apical pre-contrast short axis T2 maps were generated, using a single shot TrueFisp acquisition after different T2 preparation times (0,25,50 ms), one breathhold per slice, ECG triggered with voxel size of 2.00x 2.00 x 8 mm. The same three slices were used for stress perfusion CMR 90 seconds after hyperemia induced by regadenoson (400 mcg bolus) using 0.05 mmol/kg of gadolinium (Gadovist, Bayer Schering Pharma AG, Berlin, Germany) followed by 20ml saline flush injected at 5ml/s. Images were acquired apex to base during breath-hold at the first pass of contrast (60 measurements). A gradient echo sequence with a flip angle of 12º, bandwith 651 Hz per pixel, nonsection-selective saturation preparation, field of view 380 mm, acquisition matrix 127x160, slice thickness 10 mm, and standard motion correction was used. LGE images were acquired 10–15 minutes after intravenous administration of additional 0.15 mmol/kg of gadolinium at end diastole, using a breath-held segmented inversion-recovery steady state free precession sequence with a flip angle of 55º, a field of view 400mm, and voxel size of 1.6×1.6×8.0mm after determining the optimal inversion time (TI) using a scout sequence.

CMR interpretation was performed by one physician using commercially available software (CMR42, version 5.11, Circle Cardiovascular Imaging, Calgary, Alberta, Canada). LV wall thickness, LV mass, end diastolic volume (EDV), end systolic volume (ESV), and LV ejection fraction (EF) were measured from short axis cine images excluding papillary muscles and trabeculations. Microvascular dysfunction was considered present if a visual perfusion defect was observed. Perfusion defects were considered surrogates for ischemia. For perfusion assessment and semi-quantification, the myocardium was divided into 32 subsegments (16 American Heart association segments subdivided into an endocardial and epicardial layer). Ischemic burden for each patient was calculated based on the number of involved sub-segments, assigning 3% of myocardium to each sub-segment. Each segment was analyzed for the presence or absence of perfusion defect. Perfusion defects sparing the subendocardium and coincident with LGE were not considered, as subendocardial involvement is mandatory for microvascular dysfunction defects. The LGE was analyzed per-segment basis using a signal threshold versus reference myocardium of ≥6 standard deviation. Total LGE was expressed as a proportion of LV mass.

Native T1 and post contrast T1 values of myocardium were measured from the three slices generating T1 maps. Extracellular volume was calculated according to the previous published formula using patient haematocrit (PMID: 27899132). T2 values of myocardium were measured from the three slices generating T2 maps. Normal values for the scan used in this study are: native T1 mapping 990.66(17.5)ms and T2 mapping 47.69(4.28)ms. Normal ECV values were considered 25(3)%, as previously published (PMID: 27899132).

Three-dimensional longitudinal, circumferential, and radial strains were obtained by manually drawing epicardial and endocardial contours on the end-diastolic frame of short-axis and long-axis images (4-chamber, 2-chamber, and 3-chamber views), using an automatic feature-tracking algorithm.

**RESULTS**

**Supplemental Table 1. Univariable linear regression for factors related with tissue characteristics.**

|  | Univariable | | |
| --- | --- | --- | --- |
|  | β-estimate | 95% confidence interval | p-value |
| Native T1 (ms) |  |  |  |
| Ischemia (% of LV)* | 12.380 | 8.390 to 16.370 | <0.001 |
| MWT (mm) | 4.181 | 2.642 to 5.720 | <0.001 |
| LV mass (g/m2) | 0.493 | 0.244 to 0.742 | <0.001 |
| Non obstructive HCM | -13.588 | -31.081 to 3.905 | 0.126 |
| Female | 12.617 | -4.274 to 29.508 | 0.141 |
| Age (years) | 0.088 | -0.477 to 0.653 | 0.757 |
| ECV (%) |  |  |  |
| Ischemia (% of LV)* | 0.010 | 0.000 to 0.010 | 0.056 |
| MWT (mm) | 0.003 | 0.001 to 0.005 | 0.002 |
| LV mass (g/m2) | 0.000 | 0.000 to 0.001 | 0.025 |
| Non obstructive HCM | -0.005 | -0.027 to 0.017 | 0.646 |
| Female | 0.004 | -0.017 to 0.025 | 0.706 |
| Age (years) | 0.000 | -0.001 to 0.001 | 0.752 |
| LGE (%of LV) |  |  |  |
| Ischemia (% of LV)* | 2.020 | 0.930 to 3.310 | <0.001 |
| MWT (mm) | 0.913 | 0.525 to 1.301 | <0.001 |
| LV mass (g/m2) | 0.111 | 0.049 to 0.172 | 0.001 |
| Non obstructive HCM | -1.464 | -5.789 to 2.861 | 0.502 |
| Female | -2.372 | -6.506 to 1.762 | 0.256 |
| Age (years) | -0.042 | -0.178 to 0.094 | 0.537 |
| T2 (ms) |  |  |  |
| Ischemia (% of LV)* | 0.300 | -0.020 to 0.610 | 0.068 |
| MWT (mm) | 0.048 | -0.073 to 0.169 | 0.435 |
| LV mass (g/m2) | 0.003 | -0.016 to 0.021 | 0.768 |
| Non obstructive HCM | -1.139 | -2.299 to 0.021 | 0.054 |
| Female | 0.021 | -0.016 to 0.058 | 0.272 |
| Age (years) | 0.021 | -0.016 to 0.058 | 0.272 |

ECV – extracellular volume; HCM – hypertrophic cardiomyopathy; LGE - late gadolinium enhancement; LV – left ventricle; MWT – maximum wall thickness. *For each 10% increment of ischemia.

**Supplemental Figure 1**. Receiver-operating characteristic curve illustrating that ischemia is associated with LGE>15% (area under the curve 0.766).


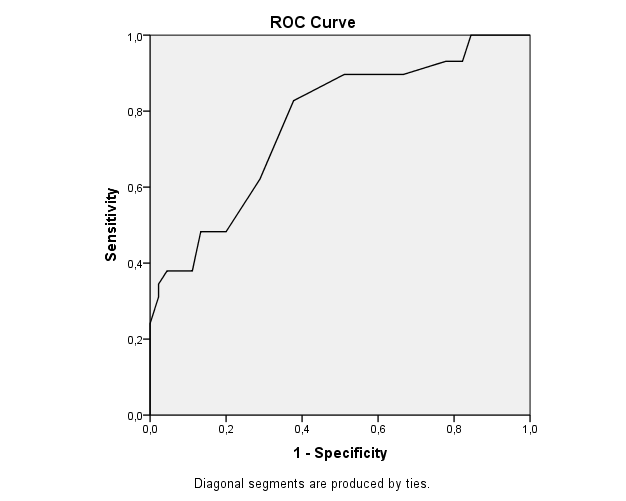


**Supplemental Table 2. Univariable and multivariable linear regression for factors related with left ventricular ejection fraction and myocardial deformation.**

|  | Univariable | | | Multivariable |  |  |
| --- | --- | --- | --- | --- | --- | --- |
|  | β-estimate | 95% confidence interval | p-value | β-estimate | 95% confidence interval | p-value |
| LVEF (%) |  |  |  |  |  |  |
| Ischemia (% of LV)* | -0.012 | -0.127 to 0.102 | 0.828 | 0.387 | -0.903 to 1.678 | 0.552 |
| MWT (mm) | -0.322 | -0.737 to 0.094 | 0.127 | -0.391 | -0.868 to 0.086 | 0.107 |
| LV mass (g/m2) | -0.053 | -0.116 to 0.009 | 0.092 |  |  |  |
| Non obstructive HCM | 2.475 | -1.604 to 6.555 | 0.230 |  |  |  |
| Male | 3.431 | -0.461 to 7.323 | 0.083 |  |  |  |
| Age (years) | 0.047 | -0.083 to 0.178 | 0.471 |  |  |  |
| Hypertension | -0.683 | -4.524 to 3.158 | 0.724 |  |  |  |
| Diabetes | -2.798 | -8.000 to 2.404 | 0.287 |  |  |  |
| Global longitudinal strain |  |  |  |  |  |  |
| Ischemia (% of LV)* | 0.085 | 0.020 to 0.151 | 0.011 | 0.337 | -0.326 to 1.001 | 0.314 |
| MWT (mm) | 0.458 | 0.232 to 0.684 | <0.001 |  |  |  |
| LV mass (g/m^2^) | 0.068 | 0.035 to 0.101 | <0.001 | 0.059 | 0.024 to 0.095 | 0.001 |
| Non obstructive HCM | -2.605 | -4.912 to -0.297 | 0.028 |  |  |  |
| Male | 0.529 | -1.773 to 2.830 | 0.648 |  |  |  |
| Age (years) | 0.017 | -0.058 to 0.093 | 0.648 |  |  |  |
| Hypertension | 1.396 | -0.816 to 3.608 | 0.212 |  |  |  |
| Diabetes | 3.185 | 0.246 to 6.123 | 0.034 |  |  |  |
| Global radial strain |  |  |  |  |  |  |
| Ischemia (% of LV)* | -0.169 | -0.304 to -0.034 | 0.015 | 0.142 | -1.091 to 1.375 | 0.819 |
| MWT (mm) | -1.309 | -1.723 to -0.895 | <0.001 | -0.674 | -1.310 to -0.038 | 0.038 |
| LV mass (g/m^2^) | -0.199 | -0.259 to -0.139 | <0.001 | -0.127 | -0.219 to -0.034 | 0.008 |
| Non obstructive HCM | 3.391 | -1.463 to 8.245 | 0.168 |  |  |  |
| Male | 4.119 | -0.530 to 8.767 | 0.082 |  |  |  |
| Age (years) | 0.123 | -0.031 to 0.277 | 0.115 |  |  |  |
| Hypertension | -3.726 | -8.249 to 0.798 | 0.105 |  |  |  |
| Diabetes | -4.693 | -10.842 to 1.456 | 0.133 |  |  |  |
| Global circumferential strain |  |  |  |  |  |  |
| Ischemia (% of LV)* | 0.050 | -0.003 to 0.103 | 0.063 | 0.062 | -0.486 to 0.610 | 0.822 |
| MWT (mm) | 0.365 | 0.187 to 0.543 | <0.001 | 0.354 | 0.154 to 0.555 | 0.001 |
| LV mass (g/m^2^) | 0.048 | 0.021 to 0.075 | 0.001 |  |  |  |
| Non obstructive HCM | -0.804 | -2.678 to 1.071 | 0.396 |  |  |  |
| Male | -1.296 | -3.089 to 0.497 | 0.154 |  |  |  |
| Age (years) | -0.012 | -0.072 to 0.048 | 0.684 |  |  |  |
| Hypertension | 0.968 | -0.782 to 2.718 | 0.274 |  |  |  |
| Diabetes | 1.992 | -0.354 to 4.339 | 0.095 |  |  |  |

HCM – hypertrophic cardiomyopathy; LV – left ventricle; LVEF – left ventricular ejection fraction; MWT – maximum wall thickness. *For each 10% increment of ischemia

**Supplemental Table 3. Univariable and multivariable analysis for factors related with electrocardiographic findings**

|  | Univariable | | | Multivariable | | |
| --- | --- | --- | --- | --- | --- | --- |
|  | OR estimate | 95% confidence interval | p-value | OR estimate | 95% confidence interval | p-value |
| LV hypertrophy |  |  |  |  |  |  |
| Ischemia (% of LV) | 1.075 | 1.018 to 1.136 | 0.010 | 1.072 | 1.013 to 1.135 | 0.017 |
| MWT (mm) | 1.107 | 0.964 to 1.271 | 0.150 |  |  |  |
| LV mass (g/m2) | 1.028 | 1.001 to 1.056 | 0.042 |  |  |  |
| Nonobstructive HCM | 0.633 | 0.181 to 2.220 | 0.475 |  |  |  |
| LV ejection fraction (%) | 1.007 | 0.942 to 1.076 | 0.843 |  |  |  |
| Native T1 (ms) | 1.025 | 1.006 to 1.045 | 0.012 |  |  |  |
| Extracellular volume (%) | 1.058 | 0.903 to 1.238 | 0.486 |  |  |  |
| T2 (ms) | 1.177 | 0.912 to 1.519 | 0.211 |  |  |  |
| LGE (% of LV) | 1.046 | 0.974 to 1.123 | 0.221 |  |  |  |
| QRS fragmentation |  |  |  |  |  |  |
| Ischemia (% of LV) | 1.005 | 0.977 to 1.035 | 0.724 |  |  |  |
| MWT (mm) | 1.053 | 0.951 to 1.167 | 0.318 |  |  |  |
| LV mass (g/m2) | 1.009 | 0.993 to 1.025 | 0.266 |  |  |  |
| Nonobstructive HCM | 1.250 | 0.447 to 3.494 | 0.670 |  |  |  |
| LV ejection fraction (%) | 0.948 | 0.893 to 1.005 | 0.074 |  |  |  |
| Native T1 (ms) | 1.007 | 0.994 to 1.021 | 0.301 |  |  |  |
| Extracellular volume (%) | 1.132 | 0.991 to 1.294 | 0.068 |  |  |  |
| T2 (ms) | 0.883 | 0.,716 to 1.090 | 0.247 |  |  |  |
| LGE (% of LV) | 1.067 | 1.002 to 1.135 | 0.042 |  |  |  |
| Q wave |  |  |  |  |  |  |
| Ischemia (% of LV) | 1.021 | 0.988 to 1.055 | 0.210 |  |  |  |
| MWT (mm) | 1.125 | 0.998 to 1.268 | 0.054 |  |  |  |
| LV mass (g/m2) | 1.016 | 0.999 to 1.034 | 0.067 |  |  |  |
| Nonobstructive HCM | 0.381 | 0.122 to 1.187 | 0.096 |  |  |  |
| LV ejection fraction (%) | 0.977 | 0.915 to 1.044 | 0.499 |  |  |  |
| Native T1 (ms) | 1.015 | 0.998 to 1.031 | 0.080 |  |  |  |
| Extracellular volume (%) | 1.162 | 0.993 to 1.359 | 0.062 |  |  |  |
| T2 (ms) | 0.982 | 0.771 to 1.251 | 0.883 |  |  |  |
| LGE (% of LV) | 1.080 | 1.009 to 1.155 | 0.027 |  |  |  |
| T wave inversion |  |  |  |  |  |  |
| Ischemia (% of LV) | 1.028 | 0.991 to 1.066 | 0.145 | 1.005 | 0.958 to 1.055 | 0.826 |
| MWT (mm) | 1.046 | 0.929 to 1.176 | 0.458 |  |  |  |
| LV mass (g/m2) | 1.009 | 0.990 to 1.028 | 0.366 |  |  |  |
| Nonobstructive HCM | 2.468 | 0.861 to 7.073 | 0.093 | 4.147 | 1.234 to 13.935 | 0.021 |
| LV ejection fraction (%) | 1.035 | 0.973 to 1.101 | 0.271 |  |  |  |
| Native T1 (ms) | 1.019 | 1.002 to 1.036 | 0.027 | 1.024 | 1.002 to 1.046 | 0.033 |
| Extracellular volume (%) | 1.112 | 0.961 to 1.287 | 0.155 |  |  |  |
| T2 mapping | 1.147 | 0.914 to 1.439 | 0.237 |  |  |  |
| LGE (% of LV) | 1.009 | 0.950 to 1.070 | 0.086 |  |  |  |

HCM – hypertrophic cardiomyopathy; LGE – late gadolinium enhancement; LV – left ventricle; MWT – maximum wall thickness; OR – Odds Ratio. *For each 10% increment of ischemia.

**Supplemental Table 4 Univariable logistic and linear regression results for factors related with arrhythmias and functional capacity.**

|  | Univariable | | |
| --- | --- | --- | --- |
|  | OR estimate | 95% confidence interval | p-value |
| Atrial fibrillation/atrial flutter |  |  |  |
| Ischemia (% of LV)* | 1.486 | 1.073 to 2.059 | 0.017 |
| Left atrial volume (ml/m^2^) | 1.093 | 1.046 to 1.143 | <0.001 |
| MWT (mm) | 1.086 | 0.965 to 1.223 | 0.171 |
| LV mass (g/m2) | 1.005 | 0.988 to 1.023 | 0.574 |
| Non obstructive HCM | 0.520 | 0.167 to 1.623 | 0.260 |
| LV ejection fraction (%) | 0.961 | 0.899 to 1.027 | 0.241 |
| Native T1 (ms) | 1.006 | 0.990 to 1.022 | 0.451 |
| Extracellular volume (%) | 1.140 | 0.982 to 1.323 | 0.084 |
| LGE (% of LV) | 1.042 | 0.978 to 1.111 | 0.202 |
| Female | 0.486 | 0.140 to 1.688 | 0.256 |
| Age (years) | 1.017 | 0.978 to 1.058 | 0.392 |
| Hypertension | 1.845 | 0.594 to 5.734 | 0.290 |
| Diabetes | 3.377 | 0.903 to 12.629 | 0.071 |
| Non sustained ventricular tachycardia |  |  |  |
| Ischemia (% of LV)* | 1.103 | 0. 808 to 1.507 | 0.536 |
| MWT (mm) | 1.109 | 0.987 to 1.247 | 0.083 |
| LV mass (g/m2) | 1.017 | 1.000 to 1.035 | 0.049 |
| Non obstructive HCM | 0.223 | 0.071 to 0.695 | 0.010 |
| LV ejection fraction (%) | 0.925 | 0.864 to 0.990 | 0.025 |
| Native T1 (ms) | 1.013 | 0.997 to 1.029 | 0.104 |
| Extracellular volume (%) | 1.182 | 1.009 to 1.383 | 0.038 |
| LGE (% of LV) | 1.101 | 1.026 to 1.180 | 0.007 |
| Female | 0.893 | 0.289 to 2.756 | 0.843 |
| Age (years) | 1.057 | 1.010 to 1.106 | 0.016 |
|  | β-estimate | 95% confidence interval | p-value |
| Peak VO_2_ |  |  |  |
| Ischemia (% of LV)* | -0.068 | -1.009 to 0.874 | 0.886 |
| MWT (mm) | 0.230 | -0.111 to 0.572 | 0.183 |
| LV mass (g/m2) | 0.017 | -0.035 to 0.068 | 0.526 |
| Non obstructive HCM | 0.719 | -2.733 to 4.170 | 0.679 |
| LV ejection fraction (%) | 0.007 | -0.183 to 0.198 | 0.938 |
| Native T1 (ms) | -0.024 | -0.069 to 0.022 | 0.301 |
| Extracellular volume (%) | -0.281 | -0.706 to 0.143 | 0.190 |
| LGE (% of LV) | -0.059 | -0.248 to 0.131 | 0.537 |
| Female | -7.132 | -9.940 to -4.324 | <0.001 |
| Age (years) | -0.258 | -0.346 to -0.169 | <0.001 |
| Hypertension | -3.863 | -6.938 to -0.789 | 0.015 |
| Diabetes | -6.188 | -10.334 to -2.042 | 0.004 |
| Smoker | 0.537 | -4.046 to 5.120 | 0.816 |
| Beta-blocker | -3.059 | -6.532 to 0.414 | 0.083 |
| Calcium channel blocker | -3.871 | -7.420 to -0.322 | 0.033 |
| Predicted Peak VO_2_ |  |  |  |
| Ischemia (% of LV)* | -0.655 | -3.821 to 2.511 | 0.681 |
| MWT (mm) | -0.082 | -1.247 to 1.083 | 0.889 |
| LV mass (g/m2) | 0.003 | -0.172 to 0.177 | 0.977 |
| Non obstructive HCM | 0.189 | -11.449 to 11.826 | 0.974 |
| LV ejection fraction (%) | 0.775 | 0.160 to 1.389 | 0.014 |
| Native T1 (ms) | -0.066 | -0.218 to 0.087 | 0.394 |
| Extracellular volume (%) | -1.109 | -2.572 to 0.353 | 0.135 |
| LGE (% of LV) | -0.852 | -1.458 to -0.247 | 0.006 |
| Female | 6.230 | -4.766 to 17.226 | 0.262 |
| Age (years) | 0.223 | -0.138 to 0.584 | 0.222 |
| Hypertension | 8.982 | -1.620 to 19.584 | 0.096 |
| Diabetes | -8.610 | -23.307 to 6.088 | 0.247 |
| Smoker | -8.127 | -23.440 to 7,186 | 0.293 |
| Beta-blocker | -4.248 | -16.163 to 7.667 | 0.479 |
| Calcium channel blocker | 3.486 | -8.846 to 15.818 | 0.575 |
| VE/VCO_2_ slope |  |  |  |
| Ischemia (% of LV)* | 0.215 | -0.521 to 0.951 | 0.562 |
| MWT (mm) | -0.018 | -0.289 to 0.253 | 0.896 |
| LV mass (g/m2) | -0.005 | -0.045 to 0.036 | 0.821 |
| Non obstructive HCM | -1.757 | -4.432 to 0.918 | 0.194 |
| LV ejection fraction (%) | 0.021 | -0.129 to 0.170 | 0.784 |
| Native T1 (ms) | 0.036 | 0.002 to 0.071 | 0.040 |
| Extracellular volume (%) | 0.465 | 0.152 to 0.778 | 0.004 |
| LGE (% of LV) | 0.174 | 0.032 to 0.317 | 0.017 |
| Female | 3.222 | 0.759 to 5.685 | 0.011 |
| Age (years) | 0.174 | 0.100 to 0.248 | <0.001 |
| Hypertension | 1.860 | -0.618 to 4.339 | 0.139 |
| Diabetes | 7.079 | 4.079 to 10.080 | <0.001 |
| Smoker | 1.034 | -2.551 to 4.618 | 0.567 |
| Beta-blocker | 0.720 | -2.058 to 3.498 | 0.607 |
| Calcium channel blocker | 1.593 | -1.258 to 4.444 | 0.269 |
| Time to anaerobic threshold |  |  |  |
| Ischemia (% of LV)* | -0.398 | -0.884 to 0.089 | 0.107 |
| MWT (mm) | 0.102 | -0.079 to 0.283 | 0.265 |
| LV mass (g/m2) | 0.005 | -0.023 to 0.032 | 0.730 |
| Non obstructive HCM | -1.620 | -3.400 to 0.160 | 0.074 |
| LV ejection fraction (%) | 0.006 | -0.094 to 0.107 | 0.904 |
| Native T1 (ms) | -0.010 | -0.034 to 0.013 | 0.384 |
| Extracellular volume (%) | -0.206 | -0.423 to 0.012 | 0.063 |
| LGE (% of LV) | -0.070 | -0.168 to 0.029 | 0.164 |
| Female | -2.399 | -4.039 to -0.760 | 0.005 |
| Age (years) | -0.096 | -0.148 to -0.044 | <0.001 |
| Hypertension | -1.798 | -3.445 to -0.152 | 0.033 |
| Diabetes | -3.824 | -5.950 to -1.699 | 0.001 |
| Smoker | 0.185 | -2.229 to 2.598 | 0.879 |
| Beta-blocker | -0.443 | -2.342 to 1.456 | 0.643 |
| Calcium channel blocker | -0.899 | -2.821 to 1.024 | 0.354 |
| VO_2_ at anaerobic threshold |  |  |  |
| Ischemia (% of LV)* | -0.101 | -0.606 to 0.405 | 0.692 |
| MWT (mm) | 0.080 | -0.106 to 0.266 | 0.393 |
| LV mass (g/m2) | 0.016 | -0.012 to 0.044 | 0.254 |
| Non obstructive HCM | -0.299 | -2.165 to 1.567 | 0.750 |
| LV ejection fraction (%) | 0.033 | -0.070 to 0.135 | 0.525 |
| Native T1 (ms) | -0.011 | -0.036 to 0.013 | 0.352 |
| Extracellular volume (%) | -0.125 | -0.353 to 0.102 | 0.275 |
| LGE (% of LV) | -0.008 | -0.111 to 0.095 | 0.877 |
| Female | -2.975 | -4.618 to -1.333 | 0.001 |
| Age (years) | -0.119 | -0.171 to -0.067 | <0.001 |
| Hypertension | -1.494 | -3.206 to 0.217 | 0.086 |
| Diabetes | -3.123 | -5.467 to -0.779 | 0.010 |
| Smoker | 0.605 | -1.857 to 3.067 | 0.625 |
| Beta-blocker | -1.224 | -3.148 to 0.700 | 0.208 |
| Calcium channel blocker | -0.884 | -2.890 to 1.122 | 0.382 |
| Circulatory power |  |  |  |
| Ischemia (% of LV)* | -26.216 | -223.126 to 170.694 | 0.791 |
| MWT (mm) | 67.677 | -2.868 to 138.222 | 0.060 |
| LV mass (g/m2) | 9.197 | -1.425 to 19.820 | 0.089 |
| Non obstructive HCM | 101.346 | -621.423 to 824.114 | 0.780 |
| LV ejection fraction (%) | 17.772 | -21.939 to 57.482 | 0.375 |
| Native T1 (ms) | -3.600 | -13.085 to 5.886 | 0.451 |
| Extracellular volume (%) | -72.835 | -161.734 to 16.064 | 0.106 |
| LGE (% of LV) | -26.578 | -65.800 to 12.644 | 0.181 |
| Female | -1250.970 | -1870.694 to -631.245 | <0.001 |
| Age (years) | -36.127 | -57.062 to -15.192 | 0.001 |
| Hypertension | -151.721 | -823.266 to 519.824 | 0.654 |
| Diabetes | -914.982 | -1810.499 to -19.464 | 0.045 |
| Smoker | 237.508 | -720.181 to 1195.198 | 0.622 |
| Beta-blocker | -838.710 | -1553.617 to -123.803 | 0.022 |
| Calcium channel blocker | -422.579 | -1183.887 to 338.729 | 0.272 |

HCM – hypertrophic cardiomyopathy; LGE – late gadolinium enhancement; LV – left ventricle; MWT – maximum wall thickness; OR – Odds Ratio; VCO_2_ - CO2 production; VE – ventilation; VO_2_ - Oxygen uptake. *For each 10% increment of ischemia.

**DISCUSSION**

**Supplemental Table 5.** Research studies for ischemia secondary to microvascular dysfunction in patients with hypertrophic cardiomyopathy

| Author and year | Number of patients | Modality | Circulatory parameters | Main conclusions |
| --- | --- | --- | --- | --- |
| Ismail et al, 2014 (1) | 35 | CMR (adenosine) | MBF quantified on a pixel-by-pixel basis | Resting MBF was significantly higher in the endocardium than in the epicardium that reversed with stress. Negative association between hMBF and wall thickness and a significantly lower probability of fibrosis in a segment with increasing hMBF. |
| Villa et al, 2016 (2) | 30 | CMR (adenosine) | Visual and quantitative perfusion analysis (segmental and high-resolution), MBF, MPR | Higher ischemic burden in HCM LGE patients comparing with patients without LGE. LGE is an important confounder in the assessment of the ischemic burden, which can be ameliorate by using high-resolution quantitative analysis. |
| Petersen et al, 2007 (3) | 35 | CMR (adenosine) | MBF | HCM patients had decreased hyperemic MBF/vasodilator response, mainly in the endocardium, in proportion to the hypertrophy. |
| Huang et al, 2013 (4) | 22 | CMR | Slope_max_ | HCM patients presented decreased slope_max_, which correlated negatively with wall thickness. |
| Camaioni et al, 2019 (5) | 101 | CMR (adenosine) | Quantitative perfusion mapping, MBF, MPR | Microvascular dysfunction was associated with hypertrophy and LGE. Perfusion was abnormal even in myocardial segments without LVH or LGE |
| Yin et al, 2017 (6) | 47 | CMR | T_peak_, Slope_max_, SI_peak_ | HCM patients presented abnormal perfusion even in non-LGE and non-hypertrophic segments. Perfusion impairment was associated with the severity of myocardial fibrosis and hypertrophy. |
| Raphael et al, 2016 | 33 | CMR (adenosine)  Cardiac catheterization (adenosine) | Backward compression  wave, backward expansion wave, forward compression wave, forward expansion wave, myocardial perfusion  reserve, CFR, MBF | HCM patients had lower CFR and very large backward compression wave during systole due to compressive deformation of intramyocardial  blood vessels. Severe LVOTO generates an additional deceleration wave during systole. The proportion of waves acting to accelerate coronary flow increased with hyperemia, and the magnitude of change was proportional to the myocardial perfusion reserve. |
| Kim et al, 2020 (7) | 115 | CMR (adenosine) | Myocardial perfusion ratio index  (MPRI) | Female gender, apical aneurysm, NSVT and LV mass index were associated with perfusion defects. |
| Chatzantonis et al, 2020 (8) | 20 | CMR | MyoTT | MyoTT was associated with maximal LV wall thickness, presence of LGE and LV global longitudinal. |
| Olivotto et al, 2011 (9) | 61 | PET (dipyridamole) | MBF | HCM patients with sarcomeric mutation presented more severe impairment of microvascular function. |
| Lorenzoni et al, 1998 (10) | 84 | PET (dipyridamole) | MBF, CVR | Lower hMBF was associated with higher NYHA functional class LV systolic dysfunction |
| Choudhury et al, 1997 (11) | 12 | PET (dipyridamole) | MBF, CVR | CVR was more impaired in HCM comparing with secondary LV hypertrophy |
| Camici et al, 1991 (12) | 23 | PET (dipyridamole) | MBF, CVR, coronary resistance | HCM patients presented blunted hMBF; CVR impairment was more pronounced in patients with chest pain; CVR was abnormal also in the nonhypertrophied segments. |
| Choudhury et al, 1999 (13) | 15 | PET (dipyridamole) | MBF, CFR | High dose verapamil therapy improved septal transmural dipyridamole MBF. |
| Bravo et al, 2012 (14) | 33 | PET (dipyridamole) | rMP, MBF, MFR | Maximal wall thickness was predictor for reduced peak MBF and MFR. There was no correlation between LVOTO and flow dynamics. |
| Bravo et al, 2016 (15) | 61 | PET (amonia) | TID index, MBF, CFR, Rmp | LV cavity dilatation was related to abnormal myocardial perfusion, globally impaired vasodilator flow reserve, and degree of hypertrophy. |
| Bravo et al, 2013 (16) | 47 | PET (amonia), CMR | MBF, CFR | Relationship between LGE by CMR and microvascular function by PET. |
| Sotgia et al, 2008 (17) | 34 | PET (amonia), CMR | MBF | LGE extention was inversely correlated and hMBF directly correlated with systolic thickening. In segments contiguous to delay enhacement areas, hMBF was significantly lower than in those remote from LGE. |
| Sciagrà et al, 2009 (18) | 95 | PET (dipyridamole) | MBF | Lower hMBF was associated with paroxysmal or chronic AF. |
| Cecchi et al, 2003 (19) | 51 | PET (dipyridamole) | MBF | Microvascular dysfunction was an independent predictor of clinical deterioration and death. |
| Olivotto et al, 2006 (20) | 51 | PET (dipyridamole) | MBF | Severe microvascular dysfunction was a long-term predictor of LV remodeling and systolic dysfunction. |

CFR - coronary flow reserve; CMR – cardiovascular magnetic resonance; CVR - coronary vasodilator reserve; hMBF - hyperemic myocardial blood flow; LGE – late gadolinium enhancement; LV - left ventricular; LVOTO - left ventricular outflow tract obstruction; MBF - myocardial blood flow; MFR - myocardial flow reserve; MPR - myocardial perfusion reserve; MPRI – myocardial perfusion ratio index; MyoTT - Myocardial transit-time; PET - Positron Emission Tomography; PD - posterior descending coronary artery; rBV - relative myocardial blood volume; rMP - regional myocardial perfusion; SI_peak_ - peak signal intensity; Slope_max_ - maximal upslope of time intensity curve; TID - transient ischemic dilatation; T_peak_ - time to peak

**References in the supplemental table 4**

1. Ismail TF, Hsu L-Y, Greve AM, Gonçalves C, Jabbour A, Gulati A, et al. Coronary microvascular ischemia in hypertrophic cardiomyopathy - a pixel-wise quantitative cardiovascular magnetic resonance perfusion study. J Cardiovasc Magn Reson. 2014;16(1):1–10.

2. Villa ADM, Sammut E, Zarinabad N, Carr-White G, Lee J, Bettencourt N, et al. Microvascular ischemia in hypertrophic cardiomyopathy: new insights from high-resolution combined quantification of perfusion and late gadolinium enhancement. J Cardiovasc Magn Reson. 2016;18:4.

3. Petersen SE, Jerosch-Herold M, Hudsmith LE, Robson MD, Francis JM, Doll HA, et al. Evidence for microvascular dysfunction in hypertrophic cardiomyopathy: New insights from multiparametric magnetic resonance imaging. Circulation. 2007;115(18):2418–25.

4. Huang L, Han R, Ai T, Sun Z, Bai Y, Cao Z, et al. Assessment of coronary microvascular dysfunction in hypertrophic cardiomyopathy: First-pass myocardial perfusion cardiovascular magnetic resonance imaging at 1.5 T. Clin Radiol. 2013;68(7):676–82.

5. Camaioni C, Knott KD, Augusto JB, Seraphim A, Rosmini S, Ricci F, et al. Inline perfusion mapping provides insights into the disease mechanism in hypertrophic cardiomyopathy. Heart. 2019;

6. Yin L, Xu H yan, Zheng S sheng, Zhu Y, Xiao J xi, Zhou W, et al. 3.0 T magnetic resonance myocardial perfusion imaging for semi-quantitative evaluation of coronary microvascular dysfunction in hypertrophic cardiomyopathy. Int J Cardiovasc Imaging. 2017;33(12):1949–59.

7. Kim EK, Lee SC, Chang SA, Jang SY, Kim SM, Park SJ, et al. Prevalence and clinical significance of cardiovascular magnetic resonance adenosine stress-induced myocardial perfusion defect in hypertrophic cardiomyopathy. J Cardiovasc Magn Reson. 2020;22(1):1–11.

8. Chatzantonis G, Bietenbeck M, Florian A, Meier C, Korthals D, Reinecke H, et al. “Myocardial transit-time” (MyoTT): a novel and easy-to-perform CMR parameter to assess microvascular disease. Clin Res Cardiol. 2020;109(4):488–97.

9. Olivotto I, Girolami F, Sciagr R, Ackerman MJ, Sotgia B, Bos JM, et al. Microvascular function is selectively impaired in patients with hypertrophic cardiomyopathy and sarcomere myofilament gene mutations. J Am Coll Cardiol. 2011;58(8):839–48.

10. Lorenzoni R, Gistri R, Cecchi F, Olivotto I, Chiriatti G, Elliott P, et al. Coronary vasodilator reserve is impaired in patients with hypertrophic cardiomyopathy and left ventricular dysfunction. Am Heart J. 1998;136(6):972–81.

11. Choudhury L, Rosen SD, Patel D, Nihoyannopoulos P, Camici PG. Coronary vasodilator reserve in primary and secondary left ventricular hypertrophy. A study with positron emission tomography. Eur Heart J. 1997;18(1):108–16.

12. Camici P, Chiriatti G, Lorenzoni R, Bellina RC, Gistri R, Italiani G, et al. Coronary vasodilation is impaired in both hypertrophied and nonhypertrophied myocardium of patients with hypertrophic cardiomyopathy: A study with nitrogen-13 ammonia and positron emission tomography. J Am Coll Cardiol. 1991;17(4):879–86.

13. Choudhury L, Elliott P, Rimoldi O, Ryan M, Lammertsma AA, Boyd H, et al. Transmural myocardial blood flow distribution in hypertrophic cardiomyopathy and effect of treatment. Basic Res Cardiol. 1999;94(1):49–59.

14. Bravo PE, Pinheiro A, Higuchi T, Rischpler C, Merrill J, Santaularia-Tomas M, et al. PET/CT assessment of symptomatic individuals with obstructive and nonobstructive hypertrophic cardiomyopathy. J Nucl Med. 2012;53(3):407–14.

15. Bravo PE, Tahari A, Pozios I, Luo HC, Bengel FM, Wahl RL, et al. Apparent left ventricular cavity dilatation during PET/CT in hypertrophic cardiomyopathy: Clinical predictors and potential mechanisms. J Nucl Cardiol. 2016;23(6):1304–14.

16. Bravo PE, Zimmerman SL, Luo HC, Pozios I, Rajaram M, Pinheiro A, et al. Relationship of delayed enhancement by magnetic resonance to myocardial perfusion by positron emission tomography in hypertrophic cardiomyopathy. Circ Cardiovasc Imaging. 2013;6(2):210–7.

17. Sotgia B, Sciagrà R, Olivotto I, Casolo G, Rega L, Betti I, et al. Spatial relationship between coronary microvascular dysfunction and delayed contrast enhancement in patients with hypertrophic cardiomyopathy. J Nucl Med. 2008;49(7):1090–6.

18. Sciagrà R, Sotgia B, Olivotto I, Cecchi F, Nistri S, Camici PG, et al. Relationship between atrial fibrillation and blunted hyperemic myocardial blood flow in patients with hypertrophic cardiomyopathy. J Nucl Cardiol. 2009;16(1):92–6.

19. Cecchi F, Sgalambro A, Baldi M, Sotgia B, Antoniucci D, Camici PG, et al. Microvascular dysfunction, myocardial ischemia, and progression to heart failure in patients with hypertrophic cardiomyopathy. J Cardiovasc Transl Res. 2009;2(4):452–61.

20. Olivotto I, Cecchi F, Gistri R, Lorenzoni R, Chiriatti G, Girolami F, et al. Relevance of coronary microvascular flow impairment to long-term remodeling and systolic dysfunction in hypertrophic cardiomyopathy. J Am Coll Cardiol. 2006;47(5):1043–8.
